# Opportunistic Infections in HIV-Infected Patients Differ Strongly in Frequencies and Spectra between Patients with Low CD4^+^ Cell Counts Examined Postmortem and Compensated Patients Examined Antemortem Irrespective of the HAART Era

**DOI:** 10.1371/journal.pone.0162704

**Published:** 2016-09-09

**Authors:** Marta K. Powell, Kamila Benková, Pavel Selinger, Marek Dogoši, Iva Kinkorová Luňáčková, Hana Koutníková, Jarmila Laštíková, Alena Roubíčková, Zuzana Špůrková, Lucie Laclová, Václav Eis, Josef Šach, Petr Heneberg

**Affiliations:** 1 Charles University in Prague, Third Faculty of Medicine, Prague, Czech Republic; 2 Na Bulovce Hospital, Pathological-Anatomical Department, Prague, Czech Republic; 3 Charles University in Prague, Second Faculty of Medicine, Department of Forensic Medicine, Prague, Czech Republic; 4 Charles University in Prague, First Faculty of Medicine, Department of Forensic Medicine and Toxicology, Prague, Czech Republic; 5 Bioptická laboratoř s.r.o., Plzeň, Czech Republic; 6 Teaching Hospital Královské Vinohrady, Department of Pathology, Prague, Czech Republic; Louisiana State University, UNITED STATES

## Abstract

**Objective:**

AIDS-related mortality has changed dramatically with the onset of highly active antiretroviral therapy (HAART), which has even allowed compensated HIV-infected patients to withdraw from secondary therapy directed against opportunistic pathogens. However, in recently autopsied HIV-infected patients, we observed that associations with a broad spectrum of pathogens remain, although detailed analyses are lacking. Therefore, we focused on the possible frequency and spectrum shifts in pathogens associated with autopsied HIV-infected patients.

**Design:**

We hypothesized that the pathogens frequency and spectrum changes found in HIV-infected patients examined postmortem did not recapitulate the changes found previously in HIV-infected patients examined antemortem in both the pre- and post-HAART eras. Because this is the first comprehensive study originating from Central and Eastern Europe, we also compared our data with those obtained in the West and Southwest Europe, USA and Latin America.

**Methods:**

We performed autopsies on 124 HIV-infected patients who died from AIDS or other co-morbidities in the Czech Republic between 1985 and 2014. The pathological findings were retrieved from the full postmortem examinations and autopsy records.

**Results:**

We collected a total of 502 host-pathogen records covering 82 pathogen species, a spectrum that did not change according to patients’ therapy or since the onset of the epidemics, which can probably be explained by the fact that even recently deceased patients were usually decompensated (in 95% of the cases, the last available CD4^+^ cell count was falling below 200 cells*μl^-1^) regardless of the treatment they received. The newly identified pathogen taxa in HIV-infected patients included *Acinetobacter calcoaceticus*, *Aerococcus viridans* and *Escherichia hermannii*. We observed a very limited overlap in both the spectra and frequencies of the pathogen species found postmortem in HIV-infected patients in Europe, the USA and Latin America.

**Conclusions:**

The shifts documented previously in compensated HIV-infected patients examined antemortem in the post-HAART era are not recapitulated in mostly decompensated HIV-infected patients examined postmortem.

## Introduction

AIDS-related mortality has changed dramatically with the onset of highly active antiretroviral therapy (HAART), which not only improved CD4^+^ cell counts in well-compensated patients but also sharply decreased the mortality rate worldwide among HIV-infected patients with fewer than 100 CD4^+^ cells*cm^-3^ [1–2]. While AIDS-associated mortality decreased by up to two orders of magnitude, non-AIDS-associated mortality decreased far less drastically, with barely over half of the numbers observed in the early 1990s [3]. Advances in the treatment of HIV-infected patients decreased the frequency of opportunistic infections, that would otherwise contribute to the mortality of patients with the most severe immunosuppression [4]. The introduction of HAART therapy allowed HIV-infected patients to withdraw from secondary therapies targeting various opportunistic pathogens, including *Pneumocystis jiroveci*, cerebral *Toxoplasma*, disseminated *Mycobacterium avium*, retinal cytomegalovirus (CMV), recurrent oroesophageal *Candida* spp., among others [5]. Secondary therapy withdrawal applies only for patients, who respond well to the HAART regimen. However, it is very likely that the spectrum and frequency of opportunistic pathogens found in HIV-infected patients in terminal stages of the disease are similar to those found in patients, who received conventional and HAART regimens. To the best of our knowledge, the data allowing for an assessment of the impact of the HAART regimen on the frequency of infection by opportunistic pathogens in deceased HIV-infected patients, particularly in those at the C3 stage, are absent, with the important exception of the recent work by Katano et al. [6]. In contrast to the clinical data obtained on compensated patients, Katano et al. reported that the total number of opportunistic pathogens found postmortem in HIV-infected persons did not change since the HAART regimen introduction, with the exception of CMV and *Pneumocystis jiroveci* infections that were approximately decreased by half in the Japanese patients cohort examined.

In this study, we hypothesized that the pathogens frequency and spectrum changes observed in HIV-infected patients examined postmortem did not recapitulate the changes found previously in HIV-infected patients examined antemortem in both the pre-HAART and post-HAART eras. Because this is the first comprehensive study originating from Central and Eastern Europe, we also compared our data with those obtained from West and Southwest Europe, the USA and Latin America (Mexico, Brazil, Peru).

## Materials and Methods

### Study population

The research cohort consisted of HIV-infected patients who died from AIDS or other co-morbidities in the Czech Republic between 1985 and 2014 and who were subjected to autopsies in any of the Prague hospitals. In total, 124 patients were subjected to autopsy, 123 at the Na Bulovce Hospital and one at the Teaching Hospital Královské Vinohrady. The cohort consisted of 104 men (mean age 42.2 ± 12.4 years) and 20 women (mean age 43.9 ± 12.7 years). The causes of death included pneumonia (36 cases), cardiorespiratory failure (24), cerebral edema (17), sepsis (9), (hepato)renal failure (7), HIV wasting syndrome (6), neoplasms (4), acute cor pulmonale (4), disseminated CMV infection (3), myocardial infarction (3), lung edema (3), hemocephalus (2), pulmonary embolism, stercoral peritonitis, duodenal bulb ulcer, septic meningitis, disseminated tuberculosis and laryngeal edema (1 case each). Among important co-morbidities were neoplasms, which included lymphoma (17 cases), lung carcinoma (2), Kaposi sarcoma, renal carcinoma, carcinoma of rectum and carcinoma of anus (1 case each). Among those, the neoplasms identified as the cause of death were lymphomas (3 cases) and a metastasizing lung carcinoma (1 case). In all cases, only a single neoplasm type (metastasized or not) was found in each patient. The detailed characteristics of patients examined in each pentad are specified in Table 1.

**Table 1:** Characteristics of patients examined in each pentad.

Pentad	Number of autopsies	Age at autopsy in years (mean ± SD [range])	Stage	Only RT monotherapy	Only RT multitherapy	HAART	Untreated
1985–1989	7	45.6 ± 13.0 [27–64]	7× C3	0	0	0	7
1990–1994	21	41.9 ± 9.4 [22–59]	21× C3	1	2	0	18
1995–1999	37	43.8 ± 15.1 [23–95]	37× C3	3	3	1	30
2000–2004	19	40.5 ± 11.5 [26–65]	1× A1, 1× A3, 1× B3, 16× C3	4	3	4	8
2005–2009	25	40.4 ± 12.2 [25–67]	2× B2, 1× B3, 1× C2, 21× C3	0	3	6	16
2010–2014	15	44.4 ± 10.6 [30–60]	1× A1, 1× B2, 2× B3, 11× C3	1	4	4	6

The first confirmed AIDS case in the Czech Republic was reported in 1985. Between 1985 and 2011, 1675 HIV-infected individuals were identified, 341 of whom developed AIDS. In total, 253 HIV-infected patients deceased, of whom 178 developed AIDS and 75 died due to other co-morbidities. Most of the HIV-infected patients were homosexual men, and sexual transmission was considered to be the key disease spreading mechanism, with only a negligible fraction of the cases associated to injection drug abuse. Over a quarter of the patients were born abroad [7].

### Experimental protocol

Pathological findings were retrieved from the full postmortem examination and autopsy records. The histopathological examination of organs and tissues included routine staining with hematoxylin and eosin. Additional stains were performed when a lesion was observed. Bacterial infections were identified by Gram staining, and bacterial species were identified by cultivation and/or polymerase chain reaction (PCR). CMV infections were identified by the presence of typical inclusion bodies in the examined tissues. The presence of CMV and other viruses was confirmed by immunohistochemistry or PCR. Tuberculosis and non-tuberculous mycobacterial infections were identified by acid-fast stain and/or PCR. Fungal and protozoal infections, including *Pneumocystis*, *Toxoplasma*, *Candida*, *Aspergillus* and *Cryptococcus*, were assessed morphologically by Grocott´s methenamine silver stain, periodic acid-Schiff stain, or immunohistochemistry. The patients’ cause of death was determined by the pathologists (authors of this manuscript or acknowledged) based on the severity, distribution, and type of illness from the autopsy or postmortem examination pathological findings. Clinical data, including age at autopsy, sex, medication and CD4^+^ cell counts at the last blood examination before death, were collected from the available medical records.

Written informed consent before the full postmortem examination was obtained from an immediate family member of the deceased patient or next of kin, as required. Informed consent was obtained in 124 cases and rejected in one case, which was excluded. Over the past three decades, informed consent forms were issued and approved by the Ethics Committees of the Na Bulovce Hospital and Teaching Hospital Královské Vinohrady. Retrospective analyses were approved by the Ethics Committee of the Third Faculty of Medicine, Charles University in Prague. The approval, which was not numbered, was issued on June 25, 2015.

### Data analyses

To analyze pathogen species richness, we computed rarefaction curves based on the log Gamma function from computing combinatorial terms, and calculated the Chao-1 estimator corrected for species unseen in the samples. We calculated basic diversity indices for each dataset. These included the total number of species found, total number of host-pathogen records, dominance (expressed as 1 –Simpson index), equitability (evenness measure), Fisher’s alpha (diversity measure), and Berger-Parker dominance index. To compare species diversitiy, we used a Shannon *t*-test with a bias correction term and the Sørensen similarity index. Following the calculation of the initial rarefaction and diversity indices involving all pathogens found, we used the Χ^2^ tests with a Bonferroni correction (controlling for the family-wise error rate) to analyze the treatment-specific deviations’ significance in species-specific infection frequencies in autopsied HIV-infected patients. We used the same test alsofor analysis of the regional differences of pathogen species-specific frequencies in HIV-infected patients. To analyze the contribution of multiple variables, we used the Canonical correspondence analysis (CCA). The CCA took into account 14 normalized variables consisting of sex, age, CDC classification of HIV infection stages transformed to two numerical values, in which the clinical category defined by letters (A to C) was transformed to numbers (1–3), and the laboratory category defined numbers (1–3) was retained but treated as a separate variable, calendar year of the patient´s death, heart complications (presence/absence), atherosclerotic vascular disease of large blood vessels (classified at a scale of severity from 0 to 3), atherosclerotic vascular disease of coronary arteries (classified at a scale of severity from 0 to 3), presence of any neoplasms, presence of particular neoplasm types (lymphoma, lung carcinoma, Kaposi carcinoma and other carcinomas), and evidence of past antiretroviral therapy. Among the variables analyzed were the particular species or higher taxa, the total counts of species of pathogens identified in each respective patient, and notification of sepsis in each respective patient. The Chao-1 estimator and Sørensen index were calculated using the EstimateS 9.1.0 software. All other calculations were performed using the PAST 2.14 software. Data are shown as the means ± SD, unless stated otherwise.

## Results

### Component community level analyses

We collected a total of 502 host-pathogen records covering 82 pathogen species. The estimated species richness, based on the Chao-1 estimator, reached 136.4 ± 25.1 species. The estimated species richness values were similar to each other for the component communities subjected to different treatment regimens, such as reverse transcriptase (RT) inhibitors in monotherapy or multitherapy, and HAART regimen. We identified 15 sepsis causing agents in HIV-infected persons. However, due to the presence of numerous doubletons and singletons within this group, the actual number of species capable of inducing sepsis in immunodeficient HIV-infected patients appears to be highly underestimated in the analyzed dataset (Table 2). The rarefaction of the total and sepsis-causing agents datasets (S1 Fig) suggested that even with a nearly complete coverage of HIV-infected patients’ autopsies performed within a single medium-sized country, we could only reach a low completeness level, particularly regarding the sepsis-inducing agents. This corresponds to relatively low Sørensen similarity index (0.42–0.57) values even if the particular diversity indices did not differ significantly from each other. An exception to this was observed when using the Shannon *t*-test to analyze the differences between the patients receiving no (or unknown) therapy and those receiving the HAART regimen. For all of the datasets analyzed, the assemblage of pathogens displayed low dominance according to the Berger-Parker dominance index, displaying values ≤0.15, and according to high equitability levels (0.81–0.94) among all the datasets analyzed (Table 2).

**Table 2:** Assessment of pathogen diversity in Czech HIV-infected subjects who underwent an autopsy between 1987 and 2014 and the pathogen distribution according to treatment regimens. This table contains the diversity indices (dominance, Fisher’s alpha, and equitability) and their comparison using Shannon diversity t-test and bootstrapping.

Diversity index	Total	Sepsis causative agents	RT inhibitiors monotherapy	RT inhibitors multitherapy	HAART and more recent	No or undisclosed therapy	*p* (RT monotherapy vs. HAART)	*p* (RT multitherapy vs. HAART)	*p* (no therapy vs. HAART)
Number of species recorded	82	15	21	28	28	71	>0.05	>0.05	>0.05
Number of host-pathogen records	502	33	34	57	60	351			
Chao-1 ± SD	136.4 ± 25.1	28.6 ± 12.9	38.7 ± 12.8	44.2 ± 11.9	39.2 ± 8.0	114.9 ± 20.8			
Dominance	0.043	0.096	0.067	0.056	0.057	0.044	>0.05	>0.05	>0.05
Brillouin	3.37	2.01	2.24	2.55	2.56	3.272			
Margalef	13.19	4.00	5.67	6.68	6.59	11.78	>0.05	>0.05	>0.05
Equitability	0.81	0.92	0.94	0.93	0.93	0.83	>0.05	>0.05	>0.05
Fisher’s alpha	28.33	10.61	23.41	21.78	20.43	26.31	>0.05	>0.05	>0.05
Berger-Parker dominance index	0.09	0.15	0.12	0.11	0.12	0.10	>0.05	>0.05	>0.05
Shannon *t*-test (*t;* d_f_; *p*)							-1.63; 72.8; >0.05	-0.08; 116.6; >0.05	4.64; 101.4; <0.001
Sørensen similarity index									
RT monotherapy vs. others			0.541	0.571	0.417				
RT multitherapy vs. others				0.545	0.536				
HAART vs. others					0.367				

### Species-specific changes

The dominant taxa included *Candida* spp. (18.7%), CMV (8.8%), *Klebsiella* spp. (8.8%), *Escherichia coli* (8.6%), *Staphylococcus* spp. (7.0%), *Pseudomonas* spp. (6.8%), *Enterococcus* spp. (6.6%) and *Acinetobacter* spp. (5.2%). No taxa displayed a frequency change in autopsied HIV-infected patients when stratified according to treatment regimens (S1 Table). Because a large proportion of patients had no medication history available, we also stratified according to the historical treatment availability timescale, separating patients autopsied during the pre-HAART era (years 1987–1995, n = 36 patients), post-HAART era (1996–2005, n = 56 patients), and second generation protease inhibitors era (2006–2014, n = 32 patients). This stratification method corroborated the result obtained when stratifying patients according to the treatment received. Except for the frequency fluctuations in *Candida* sp. infections, the infection frequencyof all analyzed taxa remained unchanged throughout the study period (S2 Table).

**Fig 1:**
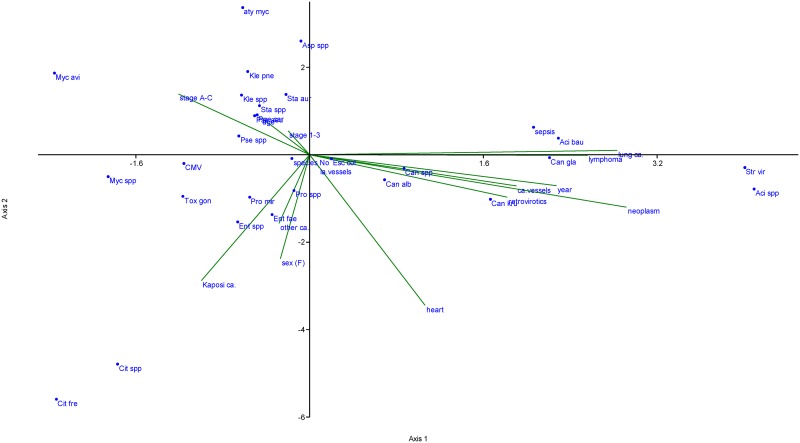
Canonical correspondence analysis of 14 normalized variables with pathogen occurrence in autopsied HIV-infected Czech patients. The variables analyzed were abbreviated according to the first letters of each respective variable and included sex, age, CDC HIV infection stage classification (abbreviated as “stage A-C” and “stage 1–3”), calendar year of the patient´s death, heart complications (presence/absence), atherosclerotic vascular disease of large blood vessels (severity scale from 0 to 3), atherosclerotic vascular disease of coronary arteries (severity scale from 0 to 3), presence of any neoplasms, presence of a particular neoplasm type (namely, lymphoma, lung carcinoma, Kaposi carcinoma and other carcinomas), and evidence of past antiretroviral therapy. Species names were abbreviated as the first three letters of the genus and species name. Alternatively the abbreviation “spp” was used when multiple species of a single genus were analyzed together. The analysis also included the species’ total counts of pathogens identified in each patient, and a binary criterion stating the presence or absence of sepsis in each patient.

The CCA (Fig 1) of normalized variables suggested that no single driver could be used as an infection predictor for a given species in an HIV-infected individual. However, the CCA revealed trends that may be worth studying further using a larger group of patients. *Klebsiella* spp. (including *Klebsiella pneumoniae*), *Mycobacterium avium*, *Staphylococcus* spp. (including *Staphylococcus aureus*) and *Aspergillus* spp. were associated with more advanced disease states (C3) and with older infected patients. *Candida* spp., particularly *Candida krusei*, were associated most frequently with more recently autopsied patients who adhered to the anti-retroviral treatment and suffered from atherosclerotic vascular disease of coronary arteries or from neoplasms (Fig 1). However, the examined cohort size did not allow for the estimation of each variable’s relative contribution.

### Records of pathogens considered to be new in HIV-infected patients

Several species identified in the course of this study represent new records in HIV-infected persons. We report an *Acinetobacter calcoaceticus* infection in the pulmonary pleurae of a cachectic 40-year-old male autopsied in 1989 (2.6 years after HIV diagnosis) and co-infected with *Pneumocystis jiroveci* and *Pseudomonas aeruginosa*. We also report the first case of an HIV-infected patient infected by a common airborne opportunistic pathogen *Aerococcus viridans*. We found this pathogen found in the esophagus, stomach, trachea, lungs, kidney, spleen, and intestine of a 39-year-old cachectic male in the C3 stage of HIV infection, whom we autopsied in 1998. This patient died from desquamative interstitial pneumonia, but also suffered from CMV-induced retinitis and epinephritis and displayed *Candida albicans* outgrowth in his stomach and intestine. Furthermore, we report one of the first cases of a human *Escherichia hermannii* infection, which is an enterobacterial species otherwise reported only in environmental sources. We found the *E*. *hermannii* infection in a cachectic 49-year-old male autopsied in 1999 (0.7 years after the HIV diagnosis), who died from respiratory failure, displayed a last CD4^+^ count 190 cells*μl^-1^, and was co-infected with *Toxoplasma gondii*, *Citrobacter freundii*, *Pseudomonas aeruginosa*, *Escherichia coli* and *Klebsiella pneumoniae*. A detailed list of all species found and the corresponding affected organs is provided in Table 3.

**Table 3:** List of the pathogens identified in HIV-infected Czech subjects autopsied between 1987 and 2014. Infection sites within the host body are indicated. Where the tissue-specific counts do not match the total counts, data on localization were unavailable in the documentation of a subset patients.

		Infection site															
Kingdom	Species	Total number of records	Sepsis	Two or more organs	Lungs	Brain	Mouth and oropharynx	Lymph nodes	Intestine and anus	Eye	Spleen	Reproductive system	Adrenal gland	Esophagus	Kidney and urinary tract	Bone	Heart
Bacteria	*Acinetobacter baumanii*	13	1	8	1			1			1				1		
Bacteria	*Acinetobacter calcoaceticus*	1			1*												
Bacteria	*Acinetobacter lwoffii*	2		1													
Bacteria	*Acinetobacter* sp.	10	2			1	2		2								
Bacteria	*Actinobacteria* gen. sp.	1															
Bacteria	*Actinomyces* sp.	1			1												
Bacteria	*Aerococcus viridans*	1		1													
Bacteria	*Alcaligenes faecalis*	1				1											
Bacteria	*Borrelia* sp.	1															
Bacteria	*Citrobacter freundii*	8		5					2								
Bacteria	*Citrobacter* sp.	4		2					2								
Bacteria	*Clostridium* sp. (non-*perfringens*)	1						1									
Bacteria	*Corynebacterium* sp.	3										1**					
Bacteria	*Corynebacterium xerosis*	1		1													
Bacteria	*Enterobacter aerogenes*	1		1													
Bacteria	*Enterobacter cloacae*	3	1	1			1										
Bacteria	*Enterobacter faecalis*	1															
Bacteria	*Enterobacter* sp.	2															
Bacteria	*Enterococcus faecalis*	29		3	1	1		2	1					1			
Bacteria	*Enterococcus faecium*	4															
Bacteria	*Escherichia coli*	41		10		2	1	1			1				1		
Bacteria	*Escherichia coli* hemolyticus	2		2													
Bacteria	*Escherichia hermannii*	1															
Bacteria	*Haemophilus parainfluenzae*	1			1												
Bacteria	*Klebsiella oxytoca*	4															
Bacteria	*Klebsiella ozaenae*	1															
Bacteria	*Klebsiella pneumoniae*	34	2	8	1		1	1						1			
Bacteria	*Klebsiella* sp.	5						1									
Bacteria	*Legionella pneumophila*	1															
Bacteria	*Listeria* sp.	1				1											
Bacteria	*Micrococcus* sp.	1															
Bacteria	*Morganella morganii*	2															
Bacteria	*Mycobacterium avium*	8	1	4	1			1									
Bacteria	*Mycobacterium kansasii*	1															
Bacteria	*Mycobacterium* sp.	8		3				2									
Bacteria	*Mycobacterium tuberculosis*	4		4													
Bacteria	*Mycobacterium xenopi*	1			1												
Bacteria	*Neisseria flavescens*	1						1									
Bacteria	*Neisseria subflava*	1									1						
Bacteria	*Proteus mirabilis*	10						1						1		1	
Bacteria	*Proteus morgani*	4															
Bacteria	*Proteus* sp.	1		1													
Bacteria	*Proteus vulgaris*	2															
Bacteria	*Pseudomonas aeruginosa*	26	1	4	2	1	1										1
Bacteria	*Pseudomonas* sp.	8				1											
Bacteria	*Rhodococcus equi*	1		1													
Bacteria	*Salmonella* group D	2				1											
Bacteria	*Salmonella* sp.	1	1														
Bacteria	*Salmonella typhimurium*	1				1											
Bacteria	*Staphylococcus aureus*	26	5	11	2	2					2						
Bacteria	*Staphylococcus capitis*	1															
Bacteria	*Staphylococcus epidermidis*	1						1									
Bacteria	*Staphylococcus haemolyticus*	1															
Bacteria	*Staphylococcus hominis*	1															
Bacteria	*Staphylococcus* sp.	5		1													
Bacteria	*Stenotrophomonas maltophilia*	1				1											
Bacteria	*Streptococcus pneumoniae*	3		1	1												
Bacteria	*Streptococcus agalactiae*	3															
Bacteria	*Streptococcus faecalis*	2															
Bacteria	*Streptococcus pyogenes*	1															
Bacteria	*Streptococcus viridans* spp.	11			1	1						1					
Bacteria	*Treponema pallidum*	1															
Bacteria	*Ureaplasma urealyticum*	1										1					
Fungi	*Aspergillus fumigatus*	1			1												
Fungi	*Aspergillus niger*	2			2												
Fungi	*Aspergillus* sp.	2			1												
Fungi	*Candida albicans*	38	5	26	2		2										
Fungi	*Candida glabrata*	12	1	8	1												
Fungi	*Candida kefyr*	1		1													
Fungi	*Candida krusei*	19	5	10			3		1								
Fungi	*Candida* sp.	21	3	8	1		3										
Fungi	*Candida tropicalis*	3		2	1												
Fungi	*Cryptococcus neoformans*	4		3					1								
Fungi	*Microsporidia* gen. sp.	1							1								
Fungi	*Penicillium marneffei*	1	1														
Fungi	*Pneumocystis jiroveci*	17	2	2	12												
Fungi	*Saccharomyces cerevisiae*	1		1													
Chromalveolata	*Toxoplasma gondii*	11			1	9						1					
Virus	cytomegalovirus	44		29	4					6			4	1			
Virus	hepatitis B virus	1															
Virus	hepatitis C virus	1															
Virus	herpes zoster & varicella simplex viruses	2															

* Pulmonary pleurae;

** Scrotum

## Discussion

The obtained data show that the pathogen frequencies and spectra associated with autopsied HIV-infected patients has not changed since the onset of the HIV epidemic. The stability of associated pathogens cannot be attributed simply to the fact that a majority of examined deceased patients did not receive any therapy. Indeed, when we stratified our HIV-infected patients according to either the known anti-retroviral therapy applied antemortem or simply according to their year of death, we did not observe nearly any significant changes. This was observed for both the component community and the species-specific level (Table 2, S1 Table). This result could be related to the fact that 118 (95%) of the cases examined in this study displayed a CD4^+^ cell count <200 cells*μl^-1^ in the last blood examination before death. Therefore, most of the deceased patients were not compensated, had severe immunosuppression, and had AIDS as a leading death-inducing factor. We also cannot exclude that the treated patients were not compliant with therapy or were not virologically suppressed. A large proportion of the previously reported improvements in the frequency of opportunistic pathogens typically associated with HIV infection (but not all of them) were based on observations made on patients under the HAART regimen, who showed a CD4^+^ cell count >200 cells*μl^-1^ [8]. By contrast, the recent study by Katano et al., who, similarly to us, examined postmortem the autopsied patients, did not observe any difference in the frequencies and spectra of pathogens associated with patients subject or not to the HAART regimen (except for lung-related illnesses, particularly cytomegalovirus infections and *Pneumocystis jiroveci* pneumonia), and they also did not observe any difference in the frequency of the particular causes of death between these two groups of patients, with the exception of cancer mortality [6].

**Fig 2:**
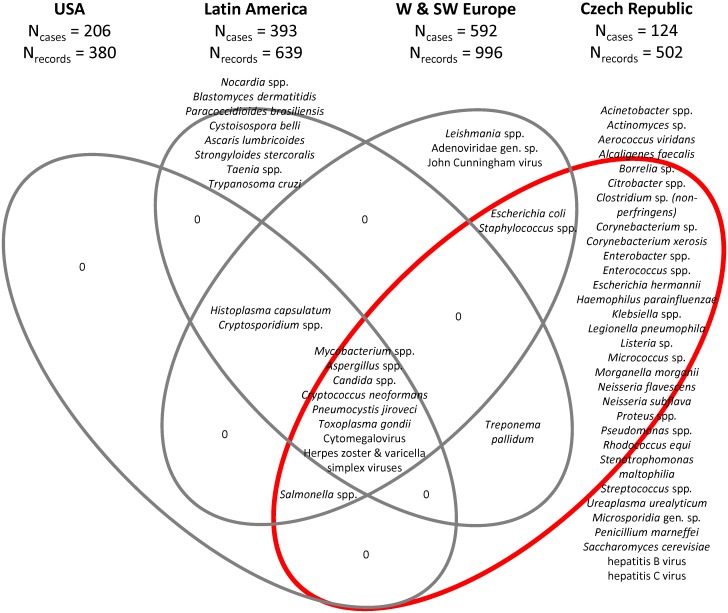
Venn diagram displaying the overlap between the pathogens identified in the autopsied HIV-infected patients included in this study (Czech Republic, Central Europe, n = 124 patients, 502 host-pathogen records), USA (n = 206 patients, 380 host-pathogen records [13–15]), Latin America (n = 393 patients, 639 host-pathogen records [16–20]) and W & SW Europe (n = 592 patients, 996 host-pathogen records [10–12]).

Importantly, the spectra and frequencies of opportunistic infections present in HIV-infected patients displays regional variation. Opportunistic pathogens may contribute to mortality particularly in countries with less developed and/or less available healthcare. This is consistent with observations made in Uganda, where 83% of autopsied HIV-infected patients died of infectious diseases. Indeed, disseminated tuberculosis was identified as a major cause of death (in 37% of cases), followed by *Cryptococcus neoformans* infection, accounting for another 20% of deaths [9]. *C*. *neoformans* infections were much less frequent in HIV-infected patients examined in our study or in the USA but the reasons for this are unknown. Of note, in this study, we did not identify any *C*. *neoformans* infections in cerebral tissues. Data on opportunistic infections in HIV-infected Central European patients are lacking. However, comparable studies were performed in both West and Southwest Europe (Britain, France and Spain [10–12]), in the USA [13–15], and in Latin America (Mexico, Brazil, Peru [16–20]). Remarkably, when comparing the pathogen spectrum reported from each of the three regions with those found in this study (Fig 2), only eight taxa were identified simultaneously in all four datasets. These consisted of the single bacterial genus, *Mycobacterium* spp., as well as of *Aspergillus* spp., *Candida* spp., *Cryptococcus neoformans*, *Pneumocystis jiroveci*, *Toxoplasma gondii*, CMV and herpetic viruses. However, even if these taxa were overlapping, they displayed a significantly different frequency within the four regions analyzed (Table 4). The only two taxa that were found in all three regions, although not in the course of this study, included *Histoplasma capsulatum* and *Cryptosporidium* spp. *Histoplasma*, a fungus commonly found in bird and bat feces, is common in HIV-infected patients in its endemic regions. However, histoplasmosis is considered to be a rare disease in Central Europe compared to other parts of the world, including OECD countries. *Cryptosporidium*, an apicomplexan, causes of diarrhea in HIV-infected patients and occurs in the Czech Republic. However, the cryptosporidiosis is frequently asymptomatic in HIV-infected patients. Therefore, cryptosporidiosis might be overlooked due to the study design used because we did not aim to profile the whole microbiome of the examined patients. Instead, we sought to identify only the pathogenic organisms associated with the lesions observed within the examined organs and tissues at time of autopsy.

**Table 4:** Differences in the frequencies of the main pathogens identified in HIV-infected subjects that underwent autopsy in the Czech Republic from 1987 to 2014 (n = 124 patients, 502 host-pathogen records, this study), USA (n = 206 patients, 380 host-pathogen records [13–15]), Latin America (n = 393 patients, 639 host-pathogen records [16–20]) and in W & SW Europe (n = 592 patients, 996 host-pathogen records [10–12]). Significance was assessed by species-specific χ^2^ tests with Bonferroni correction at n = 22.

		OBSERVED				EXPECTED				*p* (χ^2^)	
Kingdom	Species	Czech Republic	USA	Latin America	W & SW Europe	Czech Republic	USA	Latin America	W & SW Europe	Bonferroni correction: *p*<0.05 equals to *p*<2.3E-3 at n = 22	Significance of the differences observed (*** *p*<0.001, ** *p*<0.01, * *p*<0.05, n.s. = not significant)
Bacteria	*Acinetobacter* spp.	26	0	0	0	2.2	3.6	6.9	10.4	1.8E-3	*
Bacteria	*Actinomyces* sp.	1	0	0	0	0.1	0.1	0.3	0.4		
Bacteria	*Aerococcus viridans*	1	0	0	0	0.1	0.1	0.3	0.4		
Bacteria	*Alcaligenes faecalis*	1	0	0	0	0.1	0.1	0.3	0.4		
Bacteria	*Borrelia* sp.	1	0	0	0	0.1	0.1	0.3	0.4		
Bacteria	*Citrobacter* spp.	12	0	0	0	1.0	1.7	3.2	4.8	5.4E-2	n.s.
Bacteria	*Clostridium* sp. (non-*perfringens*)	1	0	0	0	0.1	0.1	0.3	0.4		
Bacteria	*Corynebacterium* sp.	3	0	0	0	0.3	0.4	0.8	1.2		
Bacteria	*Corynebacterium xerosis*	1	0	0	0	0.1	0.1	0.3	0.4		
Bacteria	*Enterobacter* spp.	7	0	0	0	0.6	1.0	1.9	2.8	1.8E-1	n.s.
Bacteria	*Enterococcus* spp.	33	0	0	0	2.8	4.6	8.7	13.2	3.2E-4	**
Bacteria	*Escherichia coli*	41	0	0	14	4.6	7.6	14.6	21.9	4.0E-6	***
Bacteria	*Escherichia hermannii*	1	0	0	0	0.1	0.1	0.3	0.4		
Bacteria	*Haemophilus parainfluenzae*	1	0	0	0	0.1	0.1	0.3	0.4		
Bacteria	*Klebsiella* spp.	44	0	0	0	3.7	6.1	11.6	17.5	2.2E-5	***
Bacteria	*Legionella pneumophila*	1	0	0	0	0.1	0.1	0.3	0.4		
Bacteria	*Listeria* sp.	1	0	0	0	0.1	0.1	0.3	0.4		
Bacteria	*Micrococcus* sp.	1	0	0	0	0.1	0.1	0.3	0.4		
Bacteria	*Morganella morganii*	2	0	0	0	0.2	0.3	0.5	0.8		
Bacteria	*Mycobacterium* spp.	22	33	91	135	23.5	39.0	74.4	112.0	3.8E-29	***
Bacteria	*Neisseria flavescens*	1	0	0	0	0.1	0.1	0.3	0.4		
Bacteria	*Neisseria subflava*	1	0	0	0	0.1	0.1	0.3	0.4		
Bacteria	*Nocardia* spp.	0	0	1	0	0.1	0.1	0.3	0.4		
Bacteria	*Proteus* spp.	17	0	0	0	1.4	2.4	4.5	6.8	1.6E-2	n.s.
Bacteria	*Pseudomonas* spp.	34	0	0	0	2.8	4.7	9.0	13.6	2.5E-4	**
Bacteria	*Rhodococcus equi*	1	0	0	0	0.1	0.1	0.3	0.4		
Bacteria	*Salmonella* spp.	4	6	0	1	0.9	1.5	2.9	4.4	2.5E-1	n.s.
Bacteria	*Staphylococcus* spp.	35	0	0	45	6.7	11.1	21.2	31.9	9.3E-9	***
Bacteria	*Stenotrophomonas maltophilia*	1	0	0	0	0.1	0.1	0.3	0.4		
Bacteria	*Streptococcus spp*.	20	0	0	0	1.7	2.8	5.3	8.0	7.7E-3	n.s.
Bacteria	*Treponema pallidum*	1	0	3	0	0.3	0.6	1.1	1.6		
Bacteria	*Ureaplasma urealyticum*	1	0	0	0	0.1	0.1	0.3	0.4		
Fungi	*Aspergillus* spp.	5	11	10	11	3.1	5.1	9.8	14.8	1.8E-3	*
Fungi	*Blastomyces dermatitidis*	0	0	1	0	0.1	0.1	0.3	0.4		
Fungi	*Candida* spp.	94	65	60	246	38.8	64.5	123.1	185.4	1.4E-48	***
Fungi	*Cryptococcus neoformans*	4	8	26	11	4.1	6.8	13.0	19.5	1.0E-4	**
Fungi	*Histoplasma capsulatum*	0	2	33	1	3.0	5.0	9.5	14.4	2.3E-3	n.s.
Fungi	*Microsporidia* gen. sp.	1	0	0	0	0.1	0.1	0.3	0.4		
Fungi	*Paracoccidioides brasiliensis*	0	0	3	0	0.3	0.4	0.8	1.2		
Fungi	*Penicillium marneffei*	1	0	0	0	0.1	0.1	0.3	0.4		
Fungi	*Pneumocystis jiroveci*	17	64	61	83	18.8	31.2	59.5	89.7	3.1E-23	***
Fungi	*Saccharomyces cerevisiae*	1	0	0	0	0.1	0.1	0.3	0.4		
Chromalveolata	*Cryptosporidium* spp.	0	14	42	92	12.4	20.5	39.2	59.0	4.2E-15	***
Chromalveolata	*Cystoisospora belli*	0	0	1	0	0.1	0.1	0.3	0.4		
Chromalveolata	*Toxoplasma gondii*	11	12	51	106	15.0	25.0	47.6	71.8	1.8E-18	***
Excavata	*Leishmania* spp.	0	0	0	16	1.3	2.2	4.2	6.4	4.9E-2	n.s.
Excavata	*Trypanosoma cruzi*	0	0	1	0	0.1	0.1	0.3	0.4		
Animalia	*Ascaris lumbricoides*	0	0	3	0	0.3	0.4	0.8	1.2		
Animalia	*Strongyloides stercoralis*	0	0	4	0	0.3	0.6	1.1	1.6		
Animalia	*Taenia* spp.	0	0	1	0	0.1	0.1	0.3	0.4		
Virus	Adenoviridae gen. sp.	0	0	0	1	0.1	0.1	0.3	0.4		
Virus	cytomegalovirus	44	120	170	201	47.1	74.2	149.3	224.8	5.4E-56	***
Virus	hepatitis B virus	1	0	0	0	0.1	0.1	0.3	0.4		
Virus	hepatitis C virus	1	0	0	0	0.1	0.1	0.3	0.4		
Virus	herpes zoster & varicella simplex viruses	2	19	12	30	5.3	8.7	16.7	25.1	3.7E-6	***
Virus	John Cunningham virus	0	0	0	3	0.3	0.4	0.8	1.2		
N/D	unknown/undisclosed agens	0	0	28	0						

We identified two groups of pathogens identified in only one of the analyzed datasets. The first group comprised the characteristic tropical and subtropical pathogens identified in the Mexican, Brazilian and Peruvian datasets, including *Paracoccidioides brasiliensis*, *Cystoisospora belli*, *Ascaris lumbricoides*, *Strongyloides stercoralis*, *Taenia* sp. and *Trypanosoma cruzi* (Fig 2). The second group comprised several dozens of species, most of which were repeatedly found in HIV-infected patients, but only this study identified them among the datasets compared in Fig 2. These species include *Acinetobacter* spp. (common in critically ill HIV-infected persons), *Aerococcus viridans* (reported only once in a HIV-infected patient [21]), *Alcaligenes faecalis* (although this species was never reported in a HIV-infected patient, *Alcaligenes faecalis* infections are particularly commonly reported in the tropics and there is a report of *Alcaligenes* sp. in HIV-infected patients but not identified to the species level [22]), *Citrobacter* spp. (a repeatedly reported infrequent infection of HIV-infected patients), *Klebsiella* spp. (commonly associated with HIV-infected patients), *Morganella morganii* (several cases of pyomyositis were previously reported in HIV-infected patients), *Neisseria flavescens* (although recognized as a common component of oral microflora of HIV-infected patients [23], we found its outgrowth in the atrophied mesenteric lymph node of 75-year-old HIV C3 male who died due to renal failure and suffered from co-infections with *Candida albicans*, *Enterococcus faecalis* and *Escherichia coli*, and who probably represented the first documented case of a pathogenic phenotype caused by *N*. *flavescens* in a HIV-infected patient), *Proteus* spp. and *Pseudomonas* spp. (both of which are common pathogens in HIV-infected patients), *Rhodococcus equi* (HIV is a leading cause of infection by this pathogen in humans [24]), *Stenotrophomonas maltophilia* (causative agent of serious infections in immunocompromised patients), *Streptococcus* spp. (common early complication of HIV infection), *Ureaplasma urealyticum* (a large portion of HIV-infected patients are positive for this causative agent of urethritis, but they only rarely develop a disease [25]), *Penicillium marneffei* (repeatedly reported in HIV-infected patients, particularly in Southeast Asia and southern China) and *Saccharomyces cerevisiae* (occasional pathologies caused by this otherwise benign organism were previously reported from immunocompromised patients).

Several pathogens found in course of this study constitute new records for HIV-infected subjects. These include *Acinetobacter calcoaceticus*, *Aerococcus viridans*, and *Escherichia hermannii*. The Gram-negative species of the *Acinetobacter calcoaceticus-Acinetobacter baumannii* complex were reported repeatedly to cause bacteremia, wound infections, pneumonia, endocarditis, and meningitis. Acinetobacters are common residents of human skin, which thus is the most likely source of infection. Infections caused by acinetobacters are difficult to treat because of their intrinsic multidrug resistance, which was also shown for *A*. *calcoaceticus* [26]. In contrast to other species of this complex, such as *A*. *baumannii*, the infections caused by *A*. *calcoaceticus* are rare, and this species is mostly isolated from soil and water environmental samples.

Gram-positive *Aerococcus viridans*, which resembles staphylococci when stained, is usually encountered as a contaminant in clinical cultures. Two distinct main sources of *A*. *viridans* isolates are the air sampled from hospital environments and lobsters, in which *A*. *viridans* is thought to cause a lethal septicemia called gaffkemia. In healthy humans, *A*. *viridans* can be found in small amount in the upper respiratory tract and/or on the skin. However, this species is considered to be a human pathogen only in immunocompromised humans, in whom it can cause meningitis, endocarditis, bacteremia, urinary tract infection, septic arthritis, and wound infections. Some of its strains display multidrug resistance [27].

Gram-negative enterobacterium *Escherichia hermannii* is only rarely pathogenic to humans, indeed, only five cases were reported until recently, all in immunocompromised individuals [28,29]. However, *E*. *hermannii* is commonly found in the wounds and feces of homeothermic organisms [30]. Numerous *E*. *hermannii* isolates were reported from dehydrated infant formula powder manufactured in Indonesia and Malaysia and from egg yolks in Korea. There is little clinical experience with this species in terms of treatment, although cefixime was reported to be effective [29]. However, systematically collected data on this species are lacking.

### Limitations

A limitation of this study is that we were not in a position of having (or having available) systematically collected antemortem clinical history of all of the patients. Therefore, we cannot answer the question as to whether they acquired their infections and were already symptomatic before their demise. This information would otherwise shed more light on the clinical presentation of the newly reported host-pathogen interactions in HIV-infected patients. Another limitation of this study stems from the fact that it is a retrospective study. Retrospective studies may be affected by a selection bias and a misclassification of information bias and by the absence of controls for the exposure or outcome assessments. These limitations prevented the establishment of a correlation between the prevalence of particular infection agent and the exact CD4^+^ cell count, or antemortem examination outcomes. However, because the HIV diagnosis is robust and we did not exclude any HIV-infected patients examined postmortem in the participating hospitals (except for the single patient whose family rejected the informed consent), the selection and information bias in this study can be considered negligible. The third limitation of this study is related to the datasets used in the comparisons (Table 4, Fig 2). We were unable to include any African patients’ dataset. Despite the fact that postmortem studies on HIV-infected African patients exist [9,31–35], they are not sufficiently detailed to allow for a direct comparison with our and the other cohorts analyzed in this study.

## Conclusions

We demonstrated that the pathogen spectrum and frequency associated with autopsied HIV-infected Czech patients with low CD4^+^ cell counts did not change since disease onset in the early 1980s, despite the substantial improvements in treatment regimens and their associated lifespan prolongation in patients after diagnosis. Combined with previous studies, data suggest that the introduction of HAART therapy and second generation protease inhibitors allowed substantially decreased burden of infections by opportunistic pathogens affecting the patients in the earlier stages of the disease, probably because the treatment causes increase in CD4^+^ cell counts and thus improves the host defense. However, the autopsied patients mostly died in the HIV C3 stage of the disease. Therefore, both their predicted and observed susceptibility to opportunistic pathogens appeared to be unchanged compared to the group of patients treated with the first generation antiretroviral therapy. Our results confirmed the unexpected previous observations made by Katano et al. [6], who also demonstrated that except for lung-related illnesses (particularly CMV infections, and *Pneumocystis jiroveci* pneumonia), there was a lack of association between the frequency of specific pathogen species and the treatment regimen of the HIV-infected patients (i.e., whether they were treated or not with HAART). We also noticed strong regional differences in the pathogens’ spectra and frequencies, and identified several opportunistic pathogens considered to be new records in HIV-infected patients.

## Supporting Information

S1 Fig Expected cumulative number of pathogenic species in autopsied HIV-infected Czech patients, as defined by rarefaction curves. Data were analyzed separately for the total pathogens and for the sepsis cases found in autopsied HIV-infected Czech patients.
(TIFF)

S1 Table Taxon-specific responses of pathogens identified in HIV-infected Czech subjects autopsied between 1987 and 2014 according to their treatment regimens. Species identified in less than four cases were excluded from the analysis. Significance was examined using species-specific χ^2^ tests with Bonferroni correction at n = 17.
(DOCX)

S2 Table Changes in the spectrum of main pathogens identified in HIV-infected Czech subjects autopsied in the pre-HAART era (between years 1987 and 1995, n = 36 patients), post-HAART era (between 1996 and 2005, n = 56 patients) and second-generation protease inhibitors era (between 2006 and 2014, n = 32 patients). Species identified in less than four cases were excluded from the analysis. Significance was examined using species-specific χ^2^ tests with Bonferroni correction at n = 17.
(DOCX)
