# Supplementary figures and images for: Opportunistic Infections in HIV-Infected Patients Differ Strongly in Frequencies and Spectra between Patients with Low CD4+ Cell Counts Examined Postmortem and Compensated Patients Examined Antemortem Irrespective of the HAART Era
Source: PLoS One. 2016 Sep 9;11(9):e0162704. doi: 10.1371/journal.pone.0162704 (PMC5017746; doi:10.1371/journal.pone.0162704)

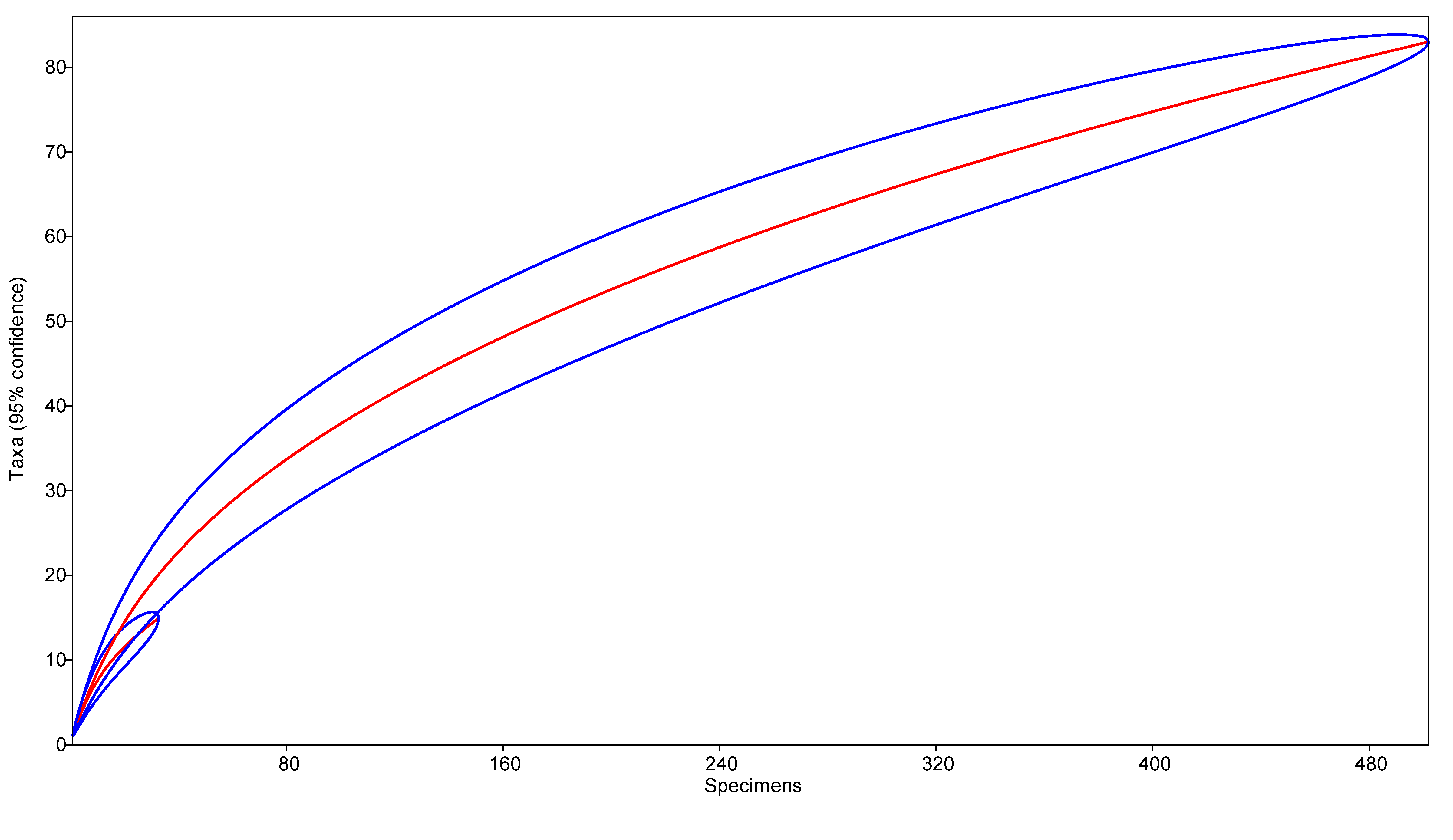

Supplement: S1 Fig — Data were analyzed separately for the total pathogens and for the sepsis cases found in autopsied HIV-infected Czech patients. (TIFF) [file pone.0162704.s001.tiff]
