# Supplementary material for: Opportunistic Infections in HIV-Infected Patients Differ Strongly in Frequencies and Spectra between Patients with Low CD4+ Cell Counts Examined Postmortem and Compensated Patients Examined Antemortem Irrespective of the HAART Era
Source: PLoS One. 2016 Sep 9;11(9):e0162704. doi: 10.1371/journal.pone.0162704 (PMC5017746; doi:10.1371/journal.pone.0162704)
Supplement: S1 Table — Species identified in less than four cases were excluded from the analysis. Significance was examined using species-specific χ2 tests with Bonferroni correction at n = 17. (DOCX) [file pone.0162704.s002.docx]

|  |  | OBSERVED | | | | EXPECTED | | | | *p* (χ^2^) | |
| --- | --- | --- | --- | --- | --- | --- | --- | --- | --- | --- | --- |
| Species | Number of records | RT inhibitiors in monotherapy | RT inhibitors in multitherapy | HAART and more recent | No or undisclosed therapy | RT inhibitiors in monotherapy | RT inhibitors in multitherapy | HAART and more recent | No or undisclosed therapy | Bonferroni correction: *p*<0.05 equals to *p*<2.9E-3 at n=17 | Significance of the differences observed (*** *p*<0.001, ** *p*<0.01, * *p*<0.05, n.s. = not significant) |
| *Candida* spp. | 94 | 8 | 12 | 13 | 61 | 6.8 | 11.4 | 11.4 | 64.4 | 8.8E-1 | n.s. |
| CMV | 44 | 4 | 4 | 1 | 35 | 3.2 | 5.3 | 5.3 | 30.2 | 1.9E-1 | n.s. |
| *Klebsiella* spp. | 44 | 3 | 6 | 5 | 30 | 3.2 | 5.3 | 5.3 | 30.2 | 9.9E-1 | n.s. |
| *Escherichia coli* | 43 | 3 | 3 | 7 | 30 | 3.0 | 5.0 | 5.0 | 28.1 | 6.3E-1 | n.s. |
| *Staphylococcus* spp. | 35 | 1 | 2 | 2 | 30 | 2.5 | 4.2 | 4.2 | 24.0 | 1.9E-1 | n.s. |
| *Pseudomonas* spp. | 34 | 2 | 1 | 5 | 26 | 2.5 | 4.1 | 4.1 | 23.3 | 4.0E-1 | n.s. |
| *Enterococcus* spp. | 33 | 4 | 7 | 5 | 17 | 2.4 | 4.0 | 4.0 | 22.6 | 1.7E-1 | n.s. |
| *Acinetobacter* spp. | 26 | 1 | 4 | 5 | 16 | 1.9 | 3.1 | 3.1 | 17.8 | 5.9E-1 | n.s. |
| *Streptococcus* spp. | 20 | 2 | 4 | 3 | 11 | 1.5 | 2.4 | 2.4 | 13.7 | 5.9E-1 | n.s. |
| *Mycobacterium* spp. | 22 | 1 | 2 | 2 | 17 | 1.6 | 2.7 | 2.7 | 15.1 | 8.5E-1 | n.s. |
| *Pneumocystis* spp. | 17 | 0 | 2 | 2 | 13 | 1.2 | 2.1 | 2.1 | 11.7 | 7.1E-1 | n.s. |
| *Proteus* spp. | 17 | 1 | 2 | 0 | 14 | 1.2 | 2.1 | 2.1 | 11.7 | 4.6E-1 | n.s. |
| *Citrobacter* spp. | 12 | 0 | 3 | 1 | 8 | 0.9 | 1.5 | 1.5 | 8.2 | 4.5E-1 | n.s. |
| *Toxoplasma gondii* | 11 | 1 | 0 | 2 | 8 | 0.8 | 1.3 | 1.3 | 7.5 | 6.3E-1 | n.s. |
| *Enterobacter* spp. | 7 | 1 | 2 | 1 | 3 | 0.5 | 0.8 | 0.8 | 4.8 | 4.3E-1 | n.s. |
| *Aspergillus* spp. | 5 | 1 | 0 | 0 | 4 | 0.4 | 0.6 | 0.6 | 3.4 | 4.9E-1 | n.s. |
| *Salmonella* spp. | 4 | 0 | 0 | 0 | 4 | 0.3 | 0.5 | 0.5 | 2.7 | 6.1E-1 | n.s. |
