# Supplementary material for: Opportunistic Infections in HIV-Infected Patients Differ Strongly in Frequencies and Spectra between Patients with Low CD4+ Cell Counts Examined Postmortem and Compensated Patients Examined Antemortem Irrespective of the HAART Era
Source: PLoS One. 2016 Sep 9;11(9):e0162704. doi: 10.1371/journal.pone.0162704 (PMC5017746; doi:10.1371/journal.pone.0162704)
Supplement: S2 Table — Species identified in less than four cases were excluded from the analysis. Significance was examined using species-specific χ2 tests with Bonferroni correction at n = 17. (DOCX) [file pone.0162704.s003.docx]

|  |  | OBSERVED | | | EXPECTED | | | *p* (χ^2^) | |
| --- | --- | --- | --- | --- | --- | --- | --- | --- | --- |
| Species | Number of records | 1987-1995 | 1996-2005 | 2006-2014 | 1987-1995 | 1996-2005 | 2006-2014 | Bonferroni correction: *p*<0.05 equals to *p*<2.9E-3 at n=17 | Significance of the differences observed (*** *p*<0.001, ** *p*<0.01, * *p*<0.05, n.s. = not significant) |
| *Candida* spp. | 94 | 13 | 59 | 22 | 27.3 | 42.5 | 24.3 | 8.5E-4 | * |
| CMV | 44 | 13 | 26 | 5 | 12.8 | 19.9 | 11.4 | 6.6E-2 | n.s. |
| *Klebsiella* spp. | 44 | 11 | 22 | 11 | 12.8 | 19.9 | 11.4 | 7.8E-1 | n.s. |
| *Escherichia coli* | 43 | 5 | 25 | 11 | 11.9 | 18.5 | 10.6 | 4.3E-2 | n.s. |
| *Staphylococcus* spp. | 35 | 7 | 21 | 7 | 10.2 | 15.8 | 9.0 | 2.1E-1 | n.s. |
| *Pseudomonas* spp. | 34 | 7 | 20 | 7 | 9.9 | 15.4 | 8.8 | 2.7E-1 | n.s. |
| *Enterococcus* spp. | 33 | 2 | 22 | 9 | 9.6 | 14.9 | 8.5 | 9.1E-3 | n.s. |
| *Acinetobacter* spp. | 26 | 4 | 16 | 6 | 7.5 | 11.7 | 6.7 | 1.9E-1 | n.s. |
| *Streptococcus* spp. | 20 | 6 | 12 | 2 | 5.8 | 9.0 | 5.2 | 2.3E-1 | n.s. |
| *Mycobacterium* spp. | 22 | 8 | 8 | 6 | 6.4 | 9.9 | 5.7 | 6.7E-1 | n.s. |
| *Pneumocystis* spp. | 17 | 5 | 4 | 8 | 4.9 | 7.7 | 4.4 | 9.4E-2 | n.s. |
| *Proteus* spp. | 17 | 7 | 7 | 3 | 4.9 | 7.7 | 4.4 | 5.1E-1 | n.s. |
| *Citrobacter* spp. | 12 | 1 | 8 | 3 | 3.5 | 5.4 | 3.1 | 4.5E-1 | n.s. |
| *Toxoplasma gondii* | 11 | 3 | 4 | 4 | 3.2 | 5.0 | 2.8 | 6.3E-1 | n.s. |
| *Enterobacter* spp. | 7 | 0 | 4 | 3 | 2.0 | 3.2 | 1.8 | 4.3E-1 | n.s. |
| *Aspergillus* spp. | 5 | 2 | 3 | 0 | 1.5 | 2.3 | 1.3 | 4.9E-1 | n.s. |
| *Salmonella* spp. | 4 | 4 | 0 | 0 | 1.2 | 1.8 | 1.0 | 6.1E-1 | n.s. |
